# Genetic Implications and Management of Epidermolysis Bullosa in the Saudi Arabian Population

**DOI:** 10.7759/cureus.66678

**Published:** 2024-08-12

**Authors:** Nancy A Shehata, Noor A Shaik, Husna Irfan Thalib

**Affiliations:** 1 Department of Dermatology, King Abdullah Medical Complex, Jeddah, SAU; 2 Department of Genetic Medicine, Faculty of Medicine at King Abdul Aziz University, Jeddah, SAU; 3 Department of General Medicine and Surgery, Batterjee Medical College, Jeddah, SAU

**Keywords:** dystrophic epidermolysis bullosa, epidermolysis bullosa simplex, junctional epidermolysis bullosa, genetic, saudi arabia

## Abstract

Epidermolysis bullosa (EB) is a genetic skin disorder characterized by skin fragility and blister formation. This review explores the genetic basis and management of EB in the Saudi population, emphasizing the need for genetic insights to enable precise diagnosis, targeted treatments, and effective counseling. Diagnosis in Saudi Arabia relies on clinical assessments and genetic testing. Prenatal diagnosis may be suggested in families with children affected by EB, but it is not widely used in the Middle East. Current management focuses on symptom relief, while emerging experimental approaches such as gene and stem cell therapies are under extensive research. Challenges in EB research include developing effective targeted therapies and understanding the variability in how genotypes manifest phenotypically. Continuous research is crucial to enhance diagnostic methods, therapeutic approaches, and overall patient care.

## Introduction and background

Inherited epidermolysis bullosa (EB) includes various skin disorders characterized by heightened skin fragility and blister formation. Globally, EB affects approximately 19.6 per million live-born infants. The exact prevalence in Middle Eastern countries, including Saudi Arabia, remains undetermined; however, it is widely prevalent due to the common practice of consanguineous marriages [1]. EB can be inherited through autosomal dominant or recessive patterns, linked to mutations in over 29 genes coding for structural proteins of the skin. These mutations impair protein function, destabilizing dermal-epidermal connections and causing blistering [2]. EB is classified into around 30 subtypes, grouped into dystrophic EB (DEB), junctional EB (JEB), EB simplex (EBS), and Kindler syndrome (KEB) based on clinical features [3].

Saudi Arabia reports few EB cases, predominantly in the Central, Eastern, and Western Provinces, often lacking detailed genetic profiling regarding involved genes, common mutations, and genotype-phenotype correlations. Research on EB in the Middle East is limited compared to other regions such as Europe, North America, and East Asia. EB significantly impacts patients' quality of life; even milder forms lead to painful blisters and wounds. Vocal cord stenosis, scarring, obstructive urethral lesions, anemia, and visual impairment are some of the potential complications. Physical and psychological challenges such as pain, social withdrawal, and skin-related embarrassment are common among EB patients [4].

## Review

Classification of EB

EB is classified into four major types based on the layer of skin affected and the clinical severity. EBS, characterized by blistering in the epidermal layer, is due to mutations in keratin genes (KRT5, KRT14). Blisters usually form within the basal layer of the epidermis. EBS varies from mild, localized forms (e.g., Weber-Cockayne type) with blisters primarily on the hands and feet, to more severe, generalized forms (e.g., Dowling-Meara type) with widespread blistering and significant morbidity. EBS is generally less severe compared to other types of EB, with symptoms often improving with age [5].

**Figure 1:**
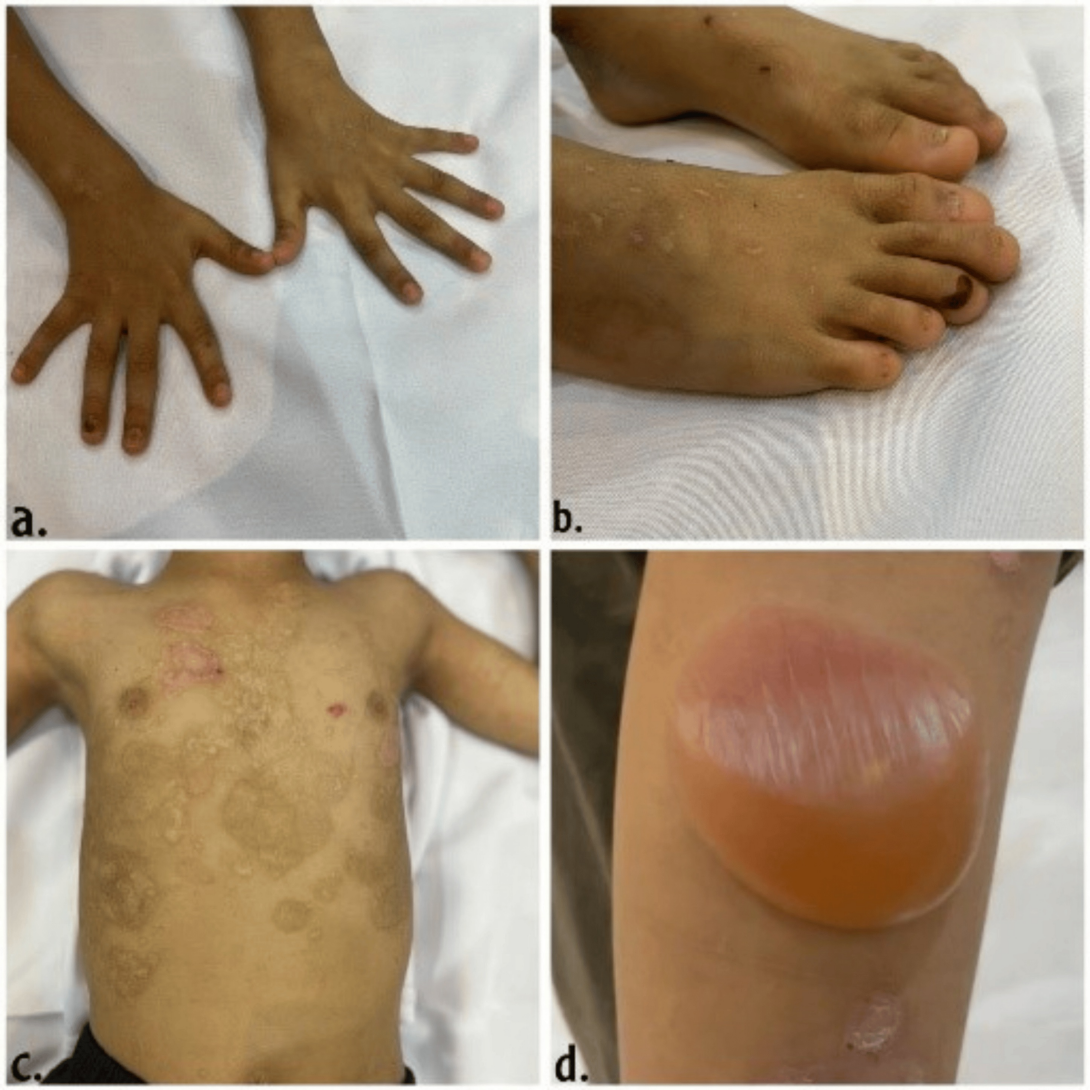
Clinical manifestations of junctional epidermolysis bullosa (JEB). Figures 1a and 1b show nail dystrophy and scar formation. Figure 1c shows hypopigmentation and hyperpigmentation with scars over the chest. Figure 1d shows new blister formation on the right arm. This figure is an original work by the authors. Formal written consent was obtained from the parents of the child.

JEB causes blistering within the lamina lucida of the basement membrane, due to mutations in genes encoding laminin-332 (LAMA3, LAMB3, LAMC2, and COL17A1). Clinical manifestations include severe skin blistering, mucosal involvement, and poor growth (Figure 1). JEB is subdivided into severe (Herlitz JEB) and non-severe forms. Herlitz JEB is often lethal in infancy due to extensive blistering, infections, and respiratory complications [6].

**Figure 2:**
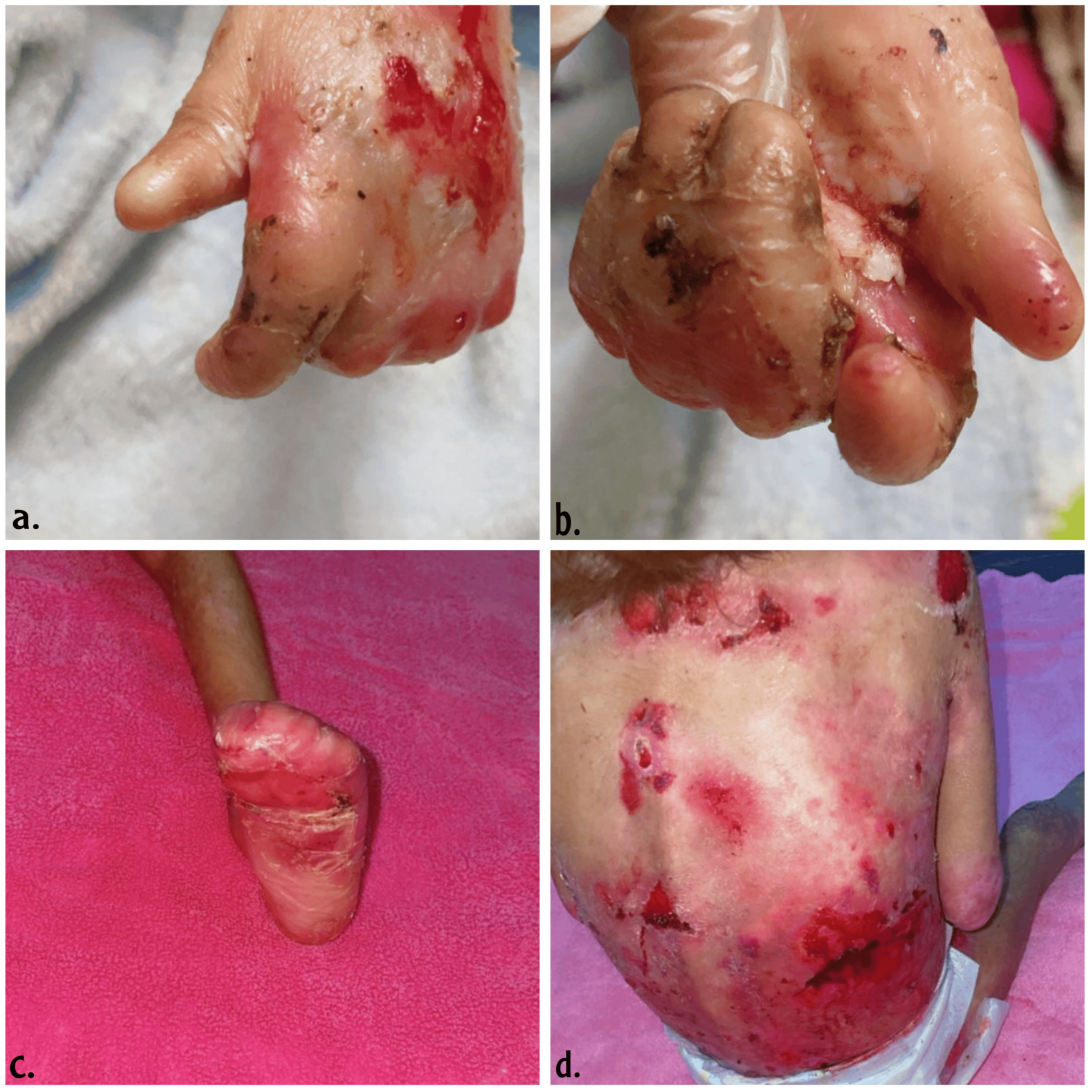
Clinical manifestations of recessive dystrophic epidermolysis bullosa. Figures 2a-2c show fusion of fingers and toes (pseudosyndactyly) and mitten deformity. Figure 2d shows inflammation, erosion, and scarring on the back. This figure is an original work of the authors. Formal written consent was obtained from the parents of the child.

**Figure 3:**
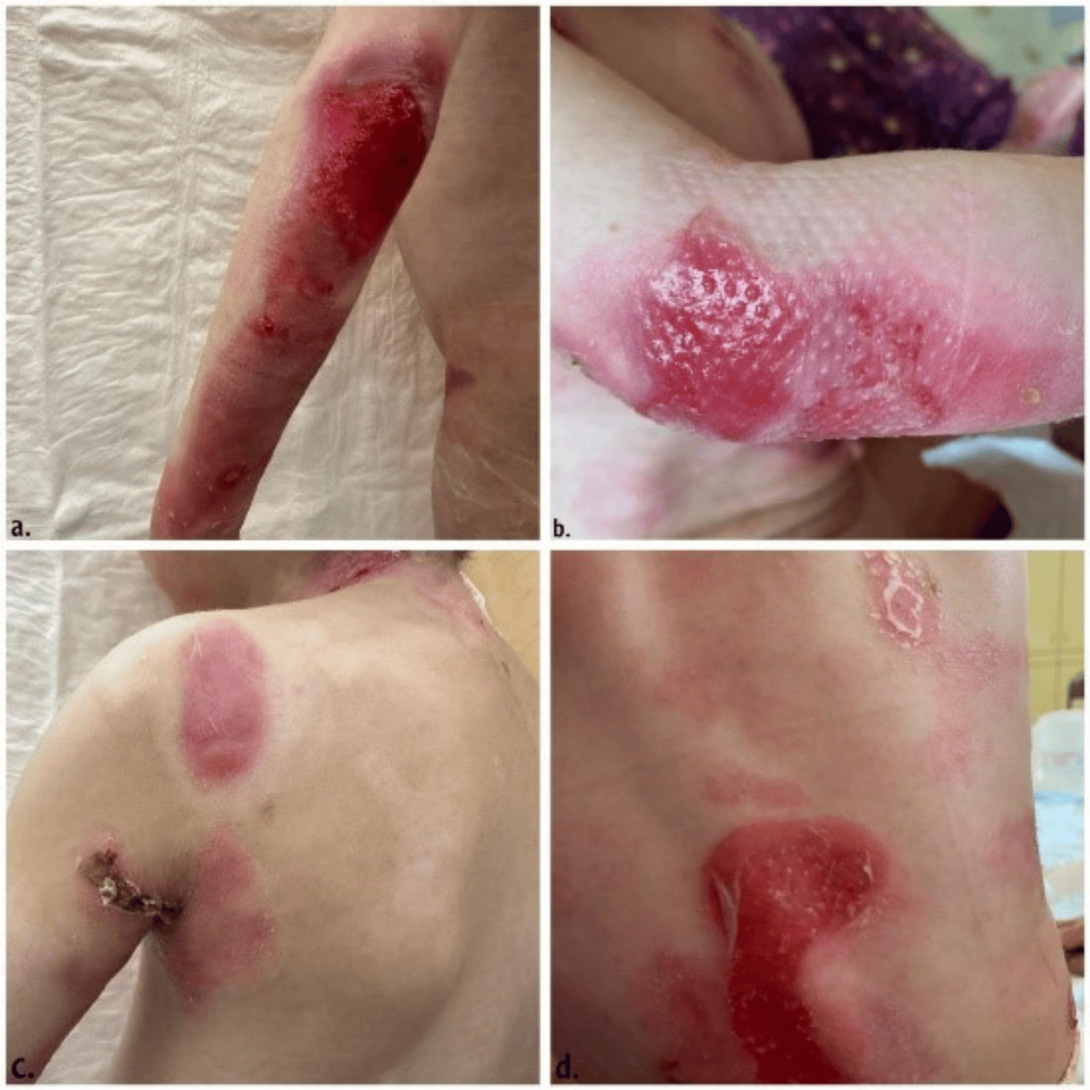
Clinical manifestations of dystrophic epidermolysis bullosa. Figures 3a and 3b show an inflamed erythematous patch with old healed scars on the arm and elbow. Figures 3c and 3d display hypopigmented scars with active erythematous patches and a newly ruptured blister with inflammation. This figure is an original work of the authors. Formal written consent was obtained from the parents of the child.

DEB results from mutations in the COL7A1 gene, leading to defective type VII collagen, which affects the anchoring fibrils that attach the dermis to the epidermis, resulting in sublamina densa blistering. DEB ranges from mild dominant forms with localized blistering and minimal scarring (DDEB) to severe recessive forms (RDEB) characterized by widespread blistering, chronic wounds, and severe scarring, often leading to significant disability, as demonstrated in Figures 2-3 [7].

KEB is a rare form of EB, caused by mutations in the FERM domain containing Kindlin 1 (FERMT1) gene, which encodes kindlin-1. This syndrome involves mixed features of EB, including blistering, photosensitivity, progressive skin atrophy, and poikiloderma. The severity of KEB varies, with few patients experiencing severe skin and mucosal involvement, leading to significant morbidity [8].

Genetic diagnostic tools for EB

Understanding the genetic basis is crucial for accurate classification, predicting disease progression, developing therapies, and providing genetic counseling [9]. EB diagnosis, as per guidelines in several countries including China, USA, UK, and Germany, involves skin sample examination using immunohistology and gene mutation analysis. Next-generation sequencing (NGS) has revolutionized EB diagnosis globally due to its accessibility and cost-effectiveness. It's useful for analyzing larger genes like COL7A1 and COL17A1, owing to NGS's ability to sequence large regions of DNA quickly and cost-effectively. In a study of 268 children from 247 families with hereditary dermatosis conducted in 2022 in Russia, NGS identified 192 pathogenic variants in 11 out of 33 genes, highlighting common mutations in COL7A1 and COL17A1 [10].

NGS gene panels can sequence multiple genes associated with a disease simultaneously, eliminating the need for pre-selecting candidate genes. This method is fast, accurate, and provides conclusive EB diagnoses. Multiple genes can cause the phenotype in EB, necessitating the testing of several genes, a mechanism known as locus heterogeneity. Multigene panel testing is a cost-effective, high-quality method, with recent panels achieving a 90% detection rate for disease-causing variants [11]. If targeted strategies fail, consider large deletions, duplications, or splice site mutations, checked by multiplex ligation-dependent probe amplification (MLPA) or PCR. RNAseq can detect splice site mutations in deep intronic regions, though it requires RNA isolation. For complex cases, whole exome sequencing (WES), clinical exome sequencing (CE), or whole genome sequencing (WGS) are the most comprehensive methods [12].

Genetic studies for EB in Saudi Arabia

A comprehensive study in the Eastern region of Saudi Arabia (1984-1990) identified sixteen cases of EB. Ten cases (62.5%) were classified as DEB, and four (25%) as EBS. Parental consanguinity was noted in fourteen cases (87.5%). All dystrophic cases had complications, prompting oral phenytoin trials in three severe cases. With nursing care and nutritional support, the treatment showed satisfactory responses. This study indicated the rarity of EB in the region and the potential effectiveness of oral phenytoin therapy combined with comprehensive care for severe cases [13]. A 2006 study at King Abdul-Aziz University, Jeddah highlighted limited research on EB in Saudi Arabia. It was the first study of EB cases from the Western province, examining 15 inherited EB cases and classifying them into three types using electron microscopy. The most common type was JEB, differing from previous reports where EBS and DEB were more prevalent, as depicted in Table 1. This emphasized the need for larger studies to understand the disease's epidemiology and characteristics locally [14].

**Table 1:** Genetic mutations in different types of EB among Saudi Arabian patients. * indicates a stop codon; p.? indicates that the predicted effect on the protein sequence is uncertain or unknown DST: Dystonin; PLEC: Plectin; TGM5: Transglutaminase 5; KRT14: Keratin 14; COL7A1: Collagen Type VII Alpha 1 Chain; LAMB3: Laminin Subunit Beta 3; EB: Epidermolysis Bullosa.

Case no.	Gene	Mutation in cDNA	Protein	Type of mutation	Clinical Presentation	Region	Ref.
Epidermolysis Bullosa Simplex							
1	DST	c.3370C>T	p.(Gln1124*)	Stop gained	Recurrent vesicles and bullae on her hands and feet. Teeth abnormality and caries	Northwestern	[16]
2	DST	c.3370C >T	p.(Gln1124*)	Stop gained	Mechanical fragility, nail abnormalities, dental caries, oral cavity erosions and lesions, and recurrent infections of the urinary and respiratory tract.	Central	[1]
3	DST	c.16496C>G	p.Al5499Gly	Missense			
4	PLEC	c.4552 C > T (NM_000445.5)	p.Gln1518*	Stop gained			
5	PLEC	c.7144C > T (NM_000445.5	p.Gln2382X*	Stop gained			
6	PLEC	c.7144C > T (NM_000445.5)	p.Gln2382X*	Stop gained			
7	TGM5	c.1335G > C (NM_004245.4)	p.Lys445Asn	Missense			
8	TGM5	c.1335G > C (NM_004245.4)	p.Lys445Asn	Missense			
9	TGM5	c.1335G > C (NM_004245.4)	p.Lys445Asn	Missense			
10	TGM5	c.1138G > C (NM_004245.4)	p.Ala380Pro	Missense			
11	KRT14	c.1094_1095delGC	p.R365LfsX117	Frameshift			
Dystrophic Epidermolysis Bullosa							
1	COL7A1	c.4199delG	G1400Vfs*310	Frameshift	Severe blistering and scarring on the hands, knees, elbows, and feet, causing pseudo-syndactyly and extensive mucosal involvement. Increased risk of aggressive squamous-cell carcinomas, gastrointestinal tract blistering, especially in the esophagus , corneal erosions and nail loss	Northern	[15]
2	COL7A1	c.1370C>T	P457L	Missense			
3	COL7A1	c.7768G > C (NM_000094.4)	p.Gly2590Arg	Missense	Mechanical fragility, nail abnormalities, dental caries, oral cavity erosions and lesions, and recurrent infections of the respiratory and urinary tracts	Central	[1]
4	COL7A1	c.7411C > T (NM_000094.4)	p.Arg2471*	Stop gained			
5	COL7A1	c.4520G > T (NM_000094.4)	p.Gly1507val	Missense			
6	COL7A1	c.4448G > A (NM_000094.4)	p.Gly1483Asp	Missense			
7	COL7A1	c.4864G > C (NM_000094.4)	p.Gly1622Arg	Missense			
8	COL7A1	c.4198delG (NM_000094.4)	G1400Vfs*310	Frameshift			
9	COL7A1	c.4198delG (NM_000094.4)	G1400Vfs*310	Frameshift			
10	COL7A1	c.4198delG (NM_000094.4)	G1400Vfs*310	Frameshift			
11	COL7A1	c.611T > G Deletion exons 25–52 (NM_000094.4)	p. Leu204Ser p.?	Missense Stop gained			
12	COL7A1	c.1507+1G > C (IVS11+1G > C) (NM_000094.4)	p.?	Splice region			
13	COL7A1	c.1507+1G > C (IVS11+1G > C) (NM_000094.4)	p.?	Splice region			
14	COL7A1	c.7442G > A (NM_000094.4)	p.?	Missense			
Junctional Epidermolysis Bullosa							
1	COL17A1	c.3922delA (NM_000494.4 )	p.Ser1308Alafs*4	Frameshift	Mechanical fragility, nail abnormalities, dental caries, oral cavity erosions and lesions, and recurrent infection of the respiratory and urinary tract.	Central	[1]
2	LAMB3	c.972delA (NM_000228.3)	p.Cys325Serfs*71	Frameshift			
3	LAMB3	c.972delA (NM_000228.3)	p.Cys325Serfs*71	Frameshift			
4	LAMB3	c.1978C > T (NM_000228.3)	p.Arg660*	Stop gained			
5	LAMB3	c.958_1034dup (NM_000228.3)	p.Asn345Lysfs*77	Frameshift			
6	LAMB3	c.1977-1G > A (NM_000228.3)	p.?	Missense			

A study in Hail included nine patients with recessive dystrophic epidermolysis bullosa (RDEB) from eight related families with common ancestry. Ages ranged from three months to seven years. Patients received treatment at the EB Center at King Khalid Hospital. Pedigree analysis was conducted using Progeny software. Skin biopsies and blood samples were analyzed at the King Faisal Specialist Hospital and Research Center. Clinical severity varied, with three patients having moderate symptoms and six severe symptoms, including extensive scars and wounds. Biopsies included punch samples from affected areas for histopathological, immunohistochemical, and electron microscopy analyses using H&E staining. Genomic DNA analysis identified two mutations in all nine patients: c.1370C>T, P457L in exon 11, and c.4199delG, G1400Vfs*310 in exon 37. The first mutation, a rare single nucleotide polymorphism (SNP), was predicted to be damaging and located in a critical domain of COL7A1. The second caused a glycine substitution and premature stop codon in the collagenous region, leading to truncated protein production. Patients presented fragile skin, blistering, and erosions with complications like infections, hair thinning, nail deformities, severe flexion contractures, and pseudosyndactyly. Regular screenings for squamous cell carcinoma and melanoma were negative, and topical antibiotics were used, with no internal organ involvement [15].

A retrospective study reviewed data from 28 Saudi EB patients at King Abdul-Aziz Medical City, Riyadh, using a custom AmpliSeq panel for 29 EB-related genes. The cohort showed a male-to-female ratio of 1.3:1, aged 3 to 21 years. Consanguinity was noted in nine patients, and family history in six. DEB was the most prevalent (42.9%), followed by EBS (35.7%) and JEB (21.4%). Mutations implicated seven genes: COL7A1 (42.9%), LAMB3 (17.9%), TGM5 (14.3%), PLEC (10.7%), DST (7%), KRT14 (3.6%), and COL17A1 (3.6%), as listed in Table 1. Patients exhibited typical EB traits: skin fragility, blisters, nail deformities, oral lesions, and infections. Genetic analysis revealed 24 mutations, 14 previously unreported. Autosomal recessive inheritance predominated (89.3%), with shared mutations observed among family members [1].

A literature review highlighted a three-year-old Saudi girl with a DST gene mutation presenting as EBS. Despite severe blistering early on, her condition did not worsen over time. Symptoms included mucous membrane erosions, onychodystrophy, hyperpigmentation, circinate acral blistering, late-onset palmoplantar keratoderma, and acral blistering. Severe EBS forms are associated with autosomal recessive PLEC gene mutations. DST gene mutations can lead to skin or neurological phenotypes. Three harmful DST mutations were identified, affecting skin and brain isoforms. Although DST anomalies are linked to neurological disorders, this patient showed no neurological symptoms. Treatment options include gene and protein replacement therapy, with genetic counseling recommended due to autosomal recessive inheritance [16].

The genetic mutations causative of EB in Saudi Arabia have been summarized in Table 1.

A 2020 review by Mariath LM et al. analyzed 171 EB cases including 49 EBS, 23 JEB, and 99 DEB. The study explored 132 pathogenic variants across eight genes to understand genotype-phenotype relationships. In EBS, congenital absence of skin (CAS) was often linked to severe forms, especially those with pyloric atresia (PA). CAS was also common in EBS cases with mutations in the kelch-like protein 24 gene. Severe EBS cases related to keratin 5 and keratin 14 gene mutations often featured CAS as a notable clinical symptom [17]. CAS is prevalent in JEB and DEB, typically due to premature termination codon variants and amino acid substitutions in integrin α6β4 genes. However, Saudi Arabian genetic studies lack detailed clinical descriptions, hindering genotype-phenotype correlations [18].

Saudi centers offering EB genetic testing include King Fahad National Guard Hospital, King Saud bin Abdulaziz University, King Abdulaziz Medical City, King Abdullah International Medical Research Center (KAIMRC), Prince Sultan Military Medical City, King Fahad Medical City, King Saud Medical City, King Khalid University Hospital, Princess Noura affiliated King Abdallah University Hospital, Al Borg Diagnostics, Centre of Excellence in Genomic Medicine Research, Medical Cells Laboratory in Jeddah, ‘Igenomix’ Genetics Laboratory in Riyadh, and Enigma Genomics Lab in Dammam [1].

Management of EB: current and future prospects

Symptomatic management involves wound care, pain management, and preventing complications. Currently, therapeutic options for EB are limited to these measures. Management in Saudi Arabia includes only dressing and topical treatment. However, recent advancements show promising potential in therapies.

Topical treatment aims to promote wound healing, reduce inflammation, and deliver gene therapy or protein replacement therapy directly to the skin. Vyjuvek (beremagene-geperpavec-svdt), a topical gene therapy for patients six months and older with DEB, reintroduces functional COL7A1 gene copies. This gene is vital for producing collagen VII, which forms anchoring fibrils connecting the dermis to the epidermis. Applied weekly by healthcare providers, the gel is mixed with a biological suspension, applied in a grid-like pattern, and covered with a dressing to enhance healing. Vyjuvek is generally well tolerated, with common side effects including itching, chills, redness, rash, cough, and runny nose. The HSV-1 vector in Vyjuvek transduces keratinocytes and fibroblasts, delivering beremagene-geperpavec-svdt into their nuclei, leading to the production of COL7 and the formation of anchoring fibrils [19]. Filsuvez (birch triterpenes), a topical gel from birch bark extract, treats partial thickness wounds in DEB and JEB patients, including children six months and older. Its exact mechanism is unknown, but the phase III EASE trial showed it significantly increased complete wound closure within 45 days. Serious side effects include allergic reactions and skin issues like hives, rash, redness, or itching. Common side effects, affecting over 2% of users, are application site reactions such as pain and itching [20]. A betulin-based oleogel from birch bark also shows anti-inflammatory properties. Research indicates that betulin aids keratinocyte differentiation. A phase 2 pilot study with DEB patients found that betulin-based oleogel supports re-epithelialization and promotes wound healing. An international Phase 3 study is ongoing to assess its safety and effectiveness in all EB types [21]. A trial (NCT01538862) examined subcutaneous granulocyte colony-stimulating factor (GCSF) injections in seven DEB patients, showing a 30% improvement in blisters, erosions, and non-healing areas [22]. Diacerein, derived from rhubarb root, is an anti-inflammatory compound that enhances skin stability in EBS. In classical EBS, mutations in keratin 5 and 14 cause cytoskeleton aggregation, leading to cell fragility and inflammation via interleukin-1β signaling. Diacerein counteracts this inflammation and stabilizes cells, as shown in laboratory tests. A phase 2/3 clinical study with Diacerein cream, albeit small and placebo-controlled, demonstrated over 40% reduction in blister counts among participants [23].

Protein replacement therapies aim to provide synthetic versions of proteins lacking in genetic diseases like EB. Experimental use of intravenous or intradermal recombinant collagen VII for DEB and Laminin-322 for JEB has shown promise in preclinical studies, but clinical trials are needed to assess effectiveness before widespread treatment [24]. Cell-based therapy, such as stem cell therapies, including mesenchymal stem cells (MSCs) from bone marrow or adipose tissue and hematopoietic stem cells (HSCs) from bone marrow or umbilical cord blood, aims to improve wound healing and reduce inflammation in EB patients [25]. Small molecule therapies target specific cellular pathways to enhance skin integrity and function. In an open-label trial (NCT02793960), coenzyme Q10 (ubidecarenone) cream was evaluated for its ability to accelerate wound healing on both affected and unaffected skin. The cream regulates cellular metabolism by targeting mitochondria, promoting the production of structural proteins crucial for skin strength. Safety is monitored through blood tests, while effectiveness is assessed using VAS pain scores and EBDASI questionnaires. Botulinum toxin, known for treating wrinkles and excessive sweating, shows promise in managing blistering and pain associated with EBS [26].

RNA-based therapies target RNA molecules to correct genetic mutations causing skin fragility in EB patients. Researchers used RNA trans-splicing to correct the RDEB phenotype in a mouse model. A SIN lentiviral vector was used to deliver a 3' RNA trans-splicing molecule replacing COL7A1 exons 65-118 in keratinocytes with a specific mutation (c.6527insC) causing type VII collagen deficiency. Corrected keratinocytes secreted type VII collagen at normal levels. Grafted onto mice, these cells robustly expressed human type VII collagen at the basement membrane zone, demonstrating the potential of this approach for clinical use [27]. Gene Therapy aims to correct genetic mutations. Early studies used retroviral gene transfer in JEB to modify patient keratinocytes, creating graftable skin equivalents in mice. While retroviral methods offer long-lasting results, they may pose cancer risks. Non-viral approaches like transposons and adeno-associated virus systems are being explored. Technologies such as CRISPR/Cas9 and TALEN also show promise in correcting EB mutations. Despite these advancements, ex vivo retroviral therapy remains attractive for its high efficiency in gene transfer to primary cells, progressing into clinical trials with precautions against oncogenic risks [28]. In the first clinical use for JEB, an adult patient's upper leg treated with retroviral-modified autologous epidermal sheets showed sustained restoration of laminin 332 expression and clinical improvement lasting over 6.5 years [29].

Subsequent successful gene therapies were reported in a 49-year-old woman and a seven-year-old boy with LAMB3 mutations. In the latter case, 80% of the patient's skin was grafted with genetically engineered sheets expressing the LAMB3 transgene, potentially saving the child’s life following a severe infection that caused significant epidermal loss. The expression of laminin 332 in the dermal-epidermal junction was maintained for up to 21 months. Analysis of keratinocyte cultures from skin biopsies indicated a gradual increase in transgene-containing holoclones, suggesting successful targeting of epidermal stem cells [30]. Gene therapy for RDEB faces the challenge of delivering the large COL7A1 gene, which encodes type VII collagen. Early efforts included direct intradermal injection of C7-expressing lentiviral vectors and protein delivery, demonstrating the potential to reverse the RDEB phenotype. A breakthrough came with the development of a modified Moloney murine leukemia virus vector for COL7A1, which successfully expressed C7 in patient keratinocytes for up to a year in preclinical studies [31].

Establishing specialized EB centers integrates dermatology, genetics, pain management, nutrition, and wound care for personalized patient care. DEBRA International supports EB patients worldwide through initiatives like EB Without Borders, aiding countries without local DEBRA support. DEBRA of America invests millions annually in patient care and research. Their advocacy efforts raise EB awareness through media collaborations and public campaigns [32]. Establishing a DEBRA chapter in KSA is crucial due to the high incidence of genetic disorders from consanguineous marriages. It would provide local patient support, medical access, educational resources, and research opportunities, enhancing awareness and improving EB management locally [33,34].

Genetic screening for carriers and counseling for EB

Once a genetic sequence variant is identified in a patient with EB, their biological parents and siblings should undergo carrier status testing at a diagnostic center, following genetic counseling guidelines. Genetic testing for EB in the Middle East may be complicated as the genetic outcome of the testing may not lead to a final conclusion. Second-hit mutations, variants of unknown significance, as well as SNPs may lead to interesting, but confusing genetic test outcomes. Understanding EB inheritance patterns is crucial. EBS is typically autosomal dominant, though rare autosomal recessive cases occur due to mutations in the K5 and K14 genes. JEB is mostly autosomal recessive due to LAMB3 gene mutations, leading to severe outcomes like neonatal mortality [35]. DEB can be inherited dominantly or recessively. Dominant DEB shows symptoms early, with localized blisters. RDEB ranges from mild (RDEB mitis) with limited symptoms to severe forms (RDEB-HS) causing extensive blistering and increased cancer risk in adults [36].

Genetic counseling is vital for families affected by EB. Typically provided by healthcare professionals specialized in medical genetics, such as physicians, genetics associates with a master’s degree in medical genetics, and medical geneticists with a PhD, these services are crucial for understanding the genetic underpinnings of EB disorders. The patient should be referred to a specific center that has the knowledge and capacity for both final genetic testing, including trio testing of and other family members, to provide the correct counseling. Genetic counseling for EB includes essential components beginning with ensuring an accurate diagnosis, which forms the foundation for all subsequent discussions and decisions. Explaining the natural progression of EB is critical, providing families with insights into expectations and future planning. Discussions include available treatment options, enabling families to make informed decisions. Clarifying the inheritance pattern, autosomal recessive, autosomal dominant, or de novo, helps assess family members' risks. Genetic counselors also facilitate referrals to specialized services and support groups, ensuring comprehensive care [36].

## Conclusions

In Saudi Arabia, the high rate of consanguineous marriages has led to a significant rise in the prevalence of EB, particularly DEB. Accurate genetic testing is crucial for guiding personalized treatments and providing thorough genetic counseling, which is essential for helping families understand their risks and make informed choices. Promising treatments like gene therapy and RNA-based therapies are being explored. The treatment of EB involves a multidisciplinary approach, including addressing the physical, emotional, and social challenges faced by patients and their families. Early diagnosis, including prenatal testing, plays a crucial role in improving care outcomes. Moving forward, research in Saudi Arabia should focus on developing new therapies for the extensive care of EB patients.
